# Micro-RNAs in Human Placenta: Tiny Molecules, Immense Power

**DOI:** 10.3390/molecules27185943

**Published:** 2022-09-13

**Authors:** Meiyuan Jin, Qiang Xu, Jiayong Li, Shouying Xu, Chao Tang

**Affiliations:** 1 National Clinical Research Center for Child Health of the Children’s Hospital, Zhejiang University School of Medicine, Hangzhou 310052, China; 2 Department of Obstetrics, Tongde Hospital of Zhejiang Province, Hangzhou 310012, China; 3 State Key Laboratory of Ophthalmology, Zhongshan Ophthalmic Center, Sun Yat-sen University, Guangzhou 510060, China

**Keywords:** micro-RNA, human pregnancy, placenta, trophoblast, gestational disorder

## Abstract

Micro-RNAs (miRNAs) are short non-coding single-stranded RNAs that modulate the expression of various target genes after transcription. The expression and distribution of kinds of miRNAs have been characterized in human placenta during different gestational stages. The identified miRNAs are recognized as key mediators in the regulation of placental development and in the maintenance of human pregnancy. Aberrant expression of miRNAs is associated with compromised pregnancies in humans, and dysregulation of those miRNAs contributes to the occurrence and development of related diseases during pregnancy, such as pre-eclampsia (PE), fetal growth restriction (FGR), gestational diabetes mellitus (GDM), recurrent miscarriage, preterm birth (PTB) and small-for-gestational-age (SGA). Thus, having a better understanding of the expression and functions of miRNAs in human placenta during pregnancy and thereby developing novel drugs targeting the miRNAs could be a potentially promising method in the prevention and treatment of relevant diseases in future. Here, we summarize the current knowledge of the expression pattern and function regulation of miRNAs in human placental development and related diseases.

## 1. Introduction

Small non-coding RNAs (ncRNAs) are a group of short non-coding RNAs that play vital roles in many biological processes and diseases [1]. Among those ncRNAs, micro-RNA (miRNA) is a small endogenous single-stranded RNA that regulates the expression of a variety of target genes after transcription. The first ncRNA was characterized in 1965 in baker’s yeast, but the first miRNA, named lin-4, was discovered by Lee et al. in 1993 [2]. As a non-coding RNA with 22 nucleotides in length, lin-4 miRNA modulates the timing of post-embryonic development through suppressing protein expression levels of lin-14 in *Caenorhabditis elegans* [2]. Later in 2000, let-7, the second miRNA, was identified in nematodes [3]. Same as lin-4 and let-7, miRNAs share a similar mechanism in function by their binding with partial complementarity towards the 3′-untranslated region (3′-UTR) of the potential target genes, respectively. To date, more than one thousand human miRNAs have been characterized so far, and each of them has the potential to control the expression of hundreds of target genes. Interestingly, while one identified characteristics of the miRNAs was the highly conserved sequences throughout species, miRNAs are distributed in a cell-dependent and tissue-specific manner [4]. Considering the nature of miRNAs, which control target gene expression but do not produce proteins themselves, it can be hypothesized that miRNAs may function as key mediators in biologically evolutionary processes and may be particularly involved in the evolution of the complexity among higher mammals. Owing to previous work in the past decades, it is now widely accepted and well believed that the tiny but powerful miRNAs function as important regulators of target genes to control and mediate various physiological or pathophysiological events among organisms including humans [5,6,7], such as the regulation of human placental development and maintenance of human pregnancy.

The placenta is a temporary but the largest organ in the fetus during pregnancy [8], while it functions as the important interface between the maternal and her fetal environments and plays a key role in health support of both the fetus as well as its mother. It is even believed that the placenta has lifelong consequences to both the fetal and maternal future well-being through various functions [9]. Firstly, the placenta participates in the exchange of multiple gases (such as oxygen), kinds of nutrients (such as amino acids) and different waste products (such as metabolic waste) between the mother and the developing fetus [10,11,12]. In addition, unlike murine placenta, the human placenta during pregnancy also functions as a major endocrine organ that produces a wide array of pregnancy-associated proteins including hormones, cytokines and growth factors to sustain fetal growth and maternal physiology, such as estradiol, progesterone and insulin growth factor 2 (IGF2) [13,14,15,16]. Moreover, the placenta serves as a natural barrier to prevent the growing fetus from being affected by the maternal immune attack of the feto–placental unit [17]. A proper placental development is indispensable to the successful pregnancy maintenance and is pivotal to embryo development, while abnormal placental development has come to be the primary defect in most major diseases associated with human pregnancy, such as pre-eclampsia (PE), gestational diabetes mellitus (GDM), fetal growth restriction (FGR), recurrent miscarriage, preterm birth (PTB) as well as small-for-gestational-age (SGA) [18,19,20,21,22,23,24].

Recently, accumulating evidence has indicated that human placental development is precisely controlled by multiple miRNAs. In both in vitro and in vivo studies using cell models and placental tissues, expression of various miRNAs is detected, whose dysregulation gives rise to the aberrant functions of placenta and is strongly associated with pregnancy-related diseases [25,26,27]. The results provide the possible evidence that treatment with molecules or drugs targeting the candidate miRNAs may contribute to the prevention of those diseases. Here, based on recent work from our and other groups, we will summarize the current stage of research on miRNAs in human placenta, and particularly we will discuss the expression, regulation and function of miRNAs in human placenta, as well as their potential involvement in human pregnancy-associated disorders.

## 2. Biogenesis and Working Mechanisms of miRNAs

**Figure 1:**
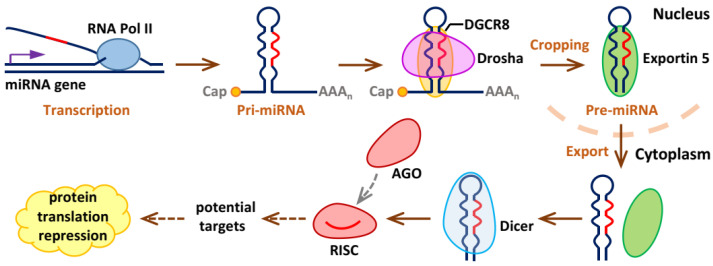
Model of canonical microRNA biogenesis.

Since their discovery in the early 1990s, large numbers of miRNAs have been characterized not only in plants but also in animals [28]. MiRNAs are a dominating class of small RNAs with ~22 nucleotides (nt) in length in almost all somatic tissues and are generated by two RNase III proteins, including Drosha and Dicer [29]. MicroRNA biogenesis initiates from transcription of a miRNA gene and comprises several post-transcriptional modifications that give rise to the maturation of miRNAs, including processing of miRNA by Drosha (a nuclear protein of ~160 kDa) and Dicer (an endonuclease of RNase III type with ~200 kDa at protein weight) in the nucleus and in the cytoplasm, respectively, argonaute protein (AGO) loading and RNA decay [30,31]. MiRNA genes are initially transcribed into long primary transcripts (pri-miRNAs) by RNA polymerase II (Pol II), and are simultaneously regulated by other molecules, such as epigenetic regulators and RNA Pol II-associated transcription factors [32]. The pri-miRNA is characterized by a local hairpin structure and miRNA sequences are embedded within this structure [33,34]. In addition, the hairpin structure is capped with 7-methylguanosine and is polyadenylated [35]. Then, by the nuclear RNase III enzyme Drosha and its co-activator DGCR8, the generated pri-miRNAs are cleaved into precursor miRNAs (pre-miRNAs), which are about 70 nt in length with a characteristic stem–loop structure that is imperfect [36,37]. Next, the produced pre-miRNAs are carried into the cytoplasm from the nucleus by protein Exportin 5 and further undergo cleavage in the presence of a second RNase III enzyme Dicer to form a miRNA duplex [38,39]. Finally, the generated RNA duplex is unwound and the miRNA acting as a guide molecule is subsequently loaded onto an AGO protein to generate an effector complex called the miRNA-induced silencing complex (RISC) for the recognition of potential targets [40] (Figure 1). Of note, RISC assembly mainly consists of two consecutive steps: (1) the loading of the RNA duplex; (2) subsequent unwinding of the RNA duplex [41].

Alterations in RNA sequence or structure will impact the maturation and turnover of miRNAs [42]. The mature miRNAs regulate gene expression at the post-transcriptional level through suppressing protein translation of target genes and/or inducing degradation of target mRNAs. MiRNA-binding sites are at most cases located in the 3′UTR of messenger RNAs (mRNAs), and the region at the 5′-end of miRNAs that spans from nucleotide position 2 to 7 is vital for their target recognition and has been termed the “seed sequence” [43,44]. Effects of mature miRNAs mainly rely on their complementary binding of the “seed sequence” to target sites located at the 3′UTR of correspondingly targeted mRNAs [38]. The nucleotide position of the seed sequence is found to be important for proper target recognition. Imperfect binding of miRNA to their partially complementary sequences located at the 3′UTRs of the targeting mRNAs gives rise to repression of subsequent protein translation; however, near-perfect binding of those miRNAs to their complementary sites within 3′UTR can result in potential cleavage and further degradation of the targeting mRNAs [45,46]. MiRNAs not only recognize 3′ UTR, some miRNAs also bind to targeting sites located at the 5′-untranslated region (5′-UTR). Recently, it has also been indicated that miRNAs negatively regulate gene expression in proliferating cells but up-regulate gene expression in quiescent cells [47]. Given that more than 60% of human protein-coding genes contain at least one conserved miRNA-binding site and that numerous non-conserved sites also exist [48], it is likely that the majority of genes coding proteins may be under the precise control of miRNAs. Thus, it is natural to think that the biogenesis and function of miRNAs are precisely regulated, and that the dysregulation of miRNAs is closely associated with human diseases.

## 3. Human Placental Development and Structure

The development of human placenta is initiated by the implantation of the blastocyst [49], and the placenta is developed from the trophectoderm (TE) [50]. During 5 days post-fertilization, the pre-implantation embryo is segregated into two different lineages, including the TE and the inner cell mass (ICM). The TE undergoes fusion and consequently forms the primary syncytium [51,52], which intrudes the maternal surface epithelium and further into the underlying maternal endometrium. Subsequently, TE cells undergo differentiation in a temporal and spatial manner and they further continue growing to intrude into the entire decidualized endometrium (decidua) up until the inner third of the myometrium as well as the maternal vasculature [53,54]. The progenitor cells of cytotrophoblasts localize at the basement membrane of multiple placental villi, and those cells thereafter differentiate along two main directions: (1) villous; and (2) extra-villous trophoblasts, both of which occur spontaneously [55,56].

Human placental villi are observed to be surrounded by a layer of mono-nucleated villous cytotrophoblasts (VCTs), which gradually undergoes fusion to form a syncytiotrophoblast (SCT) layer that is multi-nucleated. This multi-nucleated syncytium layer directly interacts with maternal blood and is fundamentally involved in the secretion and transportation of gas, nutrients and waste products across the materno–fetal interface [57,58]. The specifically multi-nucleated SCT layer also acts a key mediator in sustaining human pregnancy through regulating the secretion of pregnancy-related hormones, such as human placental lactogen (hPL), estradiol and human gonadotropin (hCG) [59]. While the non-proliferative villi interact with the maternal decidua at the tips of anchoring villi, VCT proliferates and grows to form cell columns. In the extra-villous pathway, while the trophoblast moves away from the niche of progenitors at the column base, it undergoes a process that is similar to the cellular transformation called epithelial-to-mesenchymal transition (EMT) and further differentiates into extra-villous trophoblasts (EVTs), which can be further divided into two kinds of EVTs that have distinct roles in the regulation of maternal decidua, including (1) interstitial EVTs (iEVTs); and (2) endovascular EVTs (enEVTs) [60,61]. iEVTs intrude into the maternal decidual stroma to remodel the maternal spiral arteries and continue to move as far as the maternal myometrium [62,63]. However, different from iEVTs, enEVTs, acting as a key player, move downwards inside of the spiral arteries and transiently replace the endothelium [64]. All these key steps of human placental development are precisely regulated at a temporal and spatial manner throughout pregnancy, whose disruption can cause improper development and aberrant functions of the placenta and consequently contribute to potential gestational complications.

## 4. Detection of miRNAs in Human Placenta

Owing to the detection methods such as Northern blot assays [65], quantitative real-time polymerase chain reaction (qRT-PCR) [66], microarrays [67], in situ hybridization (ISH) [68] as well as cloning of short RNAs expressed endogenously [69] and deep sequencing [70], we have obtained exciting findings in miRNA-related studies in human placenta. However, despite the merits with unique features, each method also possesses unsatisfied points. For example, Northern blot assays are able to examine the expression of miRNAs of interest and further provide useful hints on the distribution and existence of individual miRNA precursors within a specific tissue, but the sensitivity and specificity in practice are low. Although qRT-PCR provides high sensitivity in measuring miRNA expression, the throughput is not high enough, resulting in low efficiency.

MicroRNA microarray is a newly developed method with its capacity of powerful high-throughput in profiling the expression of miRNAs (particularly the large miRNAs) at the same time [71]; however, the results are always additionally needed to be verified by some other method such as qRT-PCR to make further confirmation. Different from the above methods, miRNA ISH provides with the possibility to study and observe the localization of some miRNAs of interest in specific cells or tissues; however, the sensitivity of this detection is not perfect due to its low binding affinity [72]. Nevertheless, as a powerful tool in miRNA exploration, cloning of short RNAs that are endogenously expressed is still widely accepted and utilized currently in miRNA-related studies. In addition, as a high-throughput approach for the identification and expression profiling of various miRNAs, deep sequencing enables sequencing of the entire miRNA transcriptome within each sample at a given time [73]. Thus, given the advantages and limitations in each method, ideally, more than single one method should be combined for experiments in one study, particularly in accurate miRNA expression detection, to increase the credibility and reliability of the results obtained.

## 5. Expression of Micro-RNAs in Human Placenta

It is now believed that many kinds of miRNAs can be efficiently produced by human placenta, particularly by placental trophoblasts [74]. So far, more than 500 miRNAs have been found to be expressed in human placenta [75], and Morales-Prieto et al. have reported that the expression of 762 miRNAs was successfully detected in trophoblast cells isolated from human placenta [76]. The expression of miRNAs in trophoblasts is mediated by various factors, such as hypoxia [77] (summarized in Table 1). Nowadays, hypoxia has emerged to be a common risk that may disrupt human placental development and function during pregnancy, which is associated with potentially increased incidence of gestational diseases. The expression of many miRNAs is controlled by hypoxia in human placenta [78,79,80]. For example, hypoxia up-regulates miRNA-210 (miR-210, well-known as a sensor of hypoxia) expression, which facilitates mitochondrial function in human trophoblast cells [81]. Similarly, the expression of miR-141 is induced by hypoxia and the elevated expression of miR-141 affects trophoblast cell apoptosis, invasion and vascularization [82]. Moreover, miR-218 expression is enhanced by hypoxia to suppress trophoblast cell function [83]. In addition to hypoxia, other factors including environmental factors and epigenetic modification also exert effects on the expression of miRNAs during human pregnancy [84,85,86]. Environmental factors, particularly referred to as environmental stimuli or environmental toxicants, serve as mediators in regulating placental gene expression [84]. As an endocrine disruptor group, phthalates arouse oxidative stress (OS) in human placenta. It is found that maternal exposure to phthalates results in alterations in the expression of the placental miRNA profile [87]. Similarly, mono-(2-ethylhexyl) phthalate induces miRNAs and triggers apoptosis in HTR8/SVneo cells (a kind of first trimester placental cell line), including miR-17-5p, miR-155-5p, miR-126-3p and miR-16 [88,89]. In addition, Bisphenol A (BPA), up-regulates the expression levels of several miRNAs in different trophoblast cell lines, such as miR-146a [90]. Likewise, phenol exposure is associated with alterations of miRNA expression in human placenta, including miR-142-3p, miR15a-5p and miR-185 [91]. Similar phenomena can also be observed from metals and air toxics, which are closely associated with pregnancy-related complications [92].

Epigenetic regulation, involving heritable changes in expression of genes or phenotype of cells without alterations of the underlying DNA sequence, has been shown to participate in controlling the expression of human placental miRNAs. Particularly, the status of DNA methylation has been proved to act as a key mediator in the expression of those miRNAs [93]. One famous example is the C19MC microRNA locus that is imprinted in human placenta [94]. Down-regulated C19MC methylation status is associated with the increased maternal size, which in turn, gives rise to the larger body size of the child [95].

Of note, recently, series of studies have additionally revealed that the presence of single-nucleotide polymorphism (SNP) in miRNA genes also participates in the regulation of miRNA expression and maturation during human pregnancy [96]. For example, Srivastava et al. indicated that miRNA gene polymorphism is closely associated with recurrent pregnancy loss (RPL) in a latest meta-analysis report, where 27 miRNAs with 29 different SNPs are clarified [96]. Similarly, Ezat et al. found that three different miRNA variants, including miR-126 rs4636297, miR-143 rs41291957 and miR-143 rs353292, are correlated with the risk of idiopathic RPL (iRPL) [97].

**Table 1:** Summary of factors that affect expression of miRNAs in human trophoblasts.

Factors		Affected miRNAs in Human Trophoblasts	
Hypoxia		Up-regulated	miR-210 [81], miR-141 [82], miR-218 [83]
Environment Factors	Phthalate	Up-regulated	miR-17-5p [88], miR-155-5p [88], miR-126-3p [88], miR-16 [89]
	Bisphenol A	Up-regulated	miR-146a [90]
	Phenol	Up-regulated	miR-142-3p [91], miR15a-5p [91], miR-185 [91]
Epigenetic Regulation	Methylation	Up-regulated	C19MC [95]
Single-Nucleotide Polymorphism (SNP)		-	27 miRNAs [96], miR-126 [97], miR-143 [97], miR-143 [97]

Evidence is emerging to suggest that a great deal of miRNAs are highly expressed in human placenta, and interestingly, some miRNA genes are expressed in a time-dependent and/or tissue-specific manner during different developmental stages of human placenta, which is in agreement with their functions in modulating placental development and trophoblast functions. Particularly, some miRNAs are specifically or preferentially expressed in the placenta, such as C19MC and C14MC clusters [98], which are located in chromosome 19q13.41 and 14q32, respectively. C19MC is the largest miRNA cluster identified to date, and C14MC is encoded by maternally imprinted genes. Moreover, a recent study has pointed out that C19MC also plays a key role in human placental morphogenesis [99]. Same as the miRNA clusters of C19MC and C14MC, the expression levels of some miRNAs exhibit variability within the different stages of human placental development. A previous study reveals that the expression of C19MC miRNAs in trophoblast cells is increased significantly from the first trimester to the third trimester, while intriguingly, C14MC miRNA expression presents the opposite pattern [76]. Similarly, the expression levels of the miR-371-3 cluster that is adjacent to C19MC are found to be elevated from the first to third trimester. In addition to the clusters, other miRNAs also exhibit variations in different gestational stages, such as miR-378a-5p, miR-376c and miR-371-3 [100,101,102], indicating that different miRNAs are regulated precisely and developmentally with stage-specific functions during human pregnancy.

Recent studies have clarified that kinds of miRNAs exert effects on human trophoblast cell proliferation, apoptosis, migration and invasion, suggesting human placental development is controlled by various miRNAs. In an in vitro study using the first trimester placental explants, small interfering RNA (siRNA)-mediated knockdown of Dicer significantly promotes cytotrophoblast cell proliferation and also significantly induces the expression of two molecules in pro-mitogenic signaling within cytotrophoblasts, including ERK and SHP-2 [103], indicating the role of Dicer-dependent miRNAs in regulating human early placental development. Likewise, some other miRNAs have been found to affect trophoblast cell proliferation and apoptosis. For example, miR-378a-5p promotes trophoblast cell survival by regulating Nodal [101]. Similarly, miR-376c elevates trophoblast cell proliferation by impairing transforming growth factor-β (TGF-β) and nodal signaling [100], miR-210-3p regulates trophoblast cell proliferation by fibroblast growth factor 1 (FGF1) [104], miR-133b boosts trophoblast cell proliferation by restricting SGK1 [105], and miR-518b enhances human trophoblast cell proliferation through reglating Rap1b and activating the Ras-MAPK pathway [106]. In contrast, miR-155 inhibits proliferation of human extra-villous trophoblast cells via the down-regulation of cyclin D1 expression [107], miR-424 suppresses trophoblast cell proliferation through regulating ERRγ [108], miR-184 promotes apoptosis of human trophoblast cells by targeting WIG1 [109], and miR-93 restrains trophoblast cell proliferation and induces trophoblast cell apoptosis by targeting BCL2L2 [110].

As stated above, movement (including migration and invasion) of EVTs towards the maternal decidua and myometrium are fundamental for effective placentation [111,112]. In EVTs of the human first trimester placenta, Dicer is observed to be abundantly expressed [103], suggesting miRNAs play a key role in trophoblast cell migration and invasion. A series of studies recently has uncovered a number of miRNAs that participate in regulating human trophoblast cell migration and invasion. Here are some examples: miR-34a targeting MYC inhibits human trophoblast cell invasion [113]; miR-27a suppresses trophoblast cell migration and invasion through mediating SMAD2 [114]; miR-320a negatively regulates trophoblast cell invasion via targeting estrogen-related receptor-gamma [115]; miR-431 dampens trophoblast cell migration and invasion by regulating ZEB1 [116]; miR-30a-3p modulates the invasive capacity in JEG-3 cells (a model for first trimester human trophoblasts) [117,118]; miR-7 negatively modulates human trophoblast migration and invasion by suppression of EMT [119]; miR-204 restrains human trophoblast cell invasion by targeting matrix metalloproteinase-9 (MMP-9) [120]; miR-135b down-regulates HTR-8/SVneo cell (a human trophoblastic cell line) invasion by directly inhibiting CXCL12 [121,122]; miR-145-5p inhibits HTR-8/SVneo cell invasion by down-regulation of Cyr61 [123]. In contrast, miR-378-5p enhances trophoblast cell migration and invasion via modulating Nodal [101], miR-3935 promotes human trophoblast cell migration and invasion through up-regulating EMT [124], and miR-302a potentiates trophoblast cell migration by regulating the expression levels of VEGFA [125]. Therefore, miRNAs expressed in human placenta exert both positive and negative effects on migration and invasion in trophoblast cells by regulating the expression of downstream molecules and the activity of different signaling pathways. Despite the small fraction of miRNAs that have already been characterized and investigated in migration and invasion in human trophoblast cells, further work is needed to make deeper explorations and gain a comprehensive understanding of miRNAs in regulating these cellular processes.

## 6. Micro-RNAs and Human Gestational Disorders

Aberrant expression levels of miRNAs have been both in vitro and in vivo detected in kinds of gestational complications, such as PE, FGR, GDM, and a number of studies have demonstrated the dysregulation of miRNA expression in compromised human pregnancies (summarized in Table 2).

### 6.1. Preeclampsia (PE)

PE, characterized by maternal hypertension and proteinuria, has been recognized as one of the most feared complications during human pregnancy, which negatively affects both the mother and fetus [126,127]. Many recent studies have provided the evidence that miRNAs participate in the regulation of physiological function and pathological development of placenta in pregnant women with PE. The expression of miRNAs varies between the placentas from women with PE and normal placentas and the expression of a variety of miRNAs have been observed to be increased or decreased in placentas of PE in clinic [128,129,130]. Particularly, aberrant expression of those miRNAs in human trophoblast cells gives rise to their arrested proliferation and inadequate invasion, which further lead to the failure in sufficient remodeling of maternal spiral arteries and possibly consequent deficiency in angiogenesis [74]. The miRNAs with significantly up-regulated expression in PE placenta, including miR-17 [68], miR-155 [107], miR-431 [116], miR-30a-3p [117], miR-206 [129], miR-210 [131], miR-20a [132], miR-20b [133], miR-29b [134], miR-16 [135], miR-675 [136], miR-141 [137], miR-142-3p [138], miR-125b [139], miR-200p-3b [140], miR-137 [141], miR-202-3p [142], miR-146a [143], miR-155 [144], miR-517a/b [145] and miR-517c [145], have been proved to negatively regulate trophoblast cell proliferation, migration, invasion and apoptosis. The effect of miRNAs on trophoblast cell is commonly dependent on the regulation of their corresponding target genes. For example, the up-regulated expression of miR-17 is associated with PE, and miR-17 impedes trophoblast cell function by targeting EPHB4 and ephrin-B2, which have been shown to be critical for human early placental development [68]. Similarly, in most cases, miRNA-induced alterations in trophoblast cell function such as growth and mobility rest with the expression mediation of related genes. For example, miR-125b inhibits migration and invasion of extra-villous trophoblastic cells by suppressing its target STAT3 expression, while STAT3 plays a key role in cell infiltration and vascular proliferation [139]. In addition, the up-regulated expression of miR-17~92 and miR-106a~363 clusters are demonstrated to inhibit trophoblast differentiation [146]. On the flip side, a variety of miRNAs with down-regulated expression, including miR-378a-5p [101], miR-376c [147], miR-195 [148], miR-335 [149], miR-126 [150] and miR-125a [151], exert positive effects on proliferation, migration, invasion and differentiation in human trophoblast cells, and contribute to anti-apoptosis effects. More recently, we have revealed that miR-3935, whose expression is significantly decreased in preeclamptic placental tissues, promotes human trophoblast cell EMT through regulating TRAF6-RGS2 signaling [124]. More recently, it has been suggested that miRNAs are selectively packaged into placental-derived exosomes (a kind of extracellular vesicles) and that miRNA species are differentially expressed in exosomes from women with PE and those from controls with normal pregnancy [152,153], indicating that placental exosomes may serve as mediators in the pathogenesis of PE and as early biomarkers of PE diagnosis. Given that placenta-derived exosomes participate in communication between maternal and fetal cells, it is thereby supposed that those exosomal microRNAs act as mediators in cellular biological processes including proliferation, apoptosis, invasion and migration, which have been identified to be dysregulated in PE. For example, the expression of miR-153-3p, miR-653-5p, and miR-325 is decreased in exosomes of a PE group [152], whereas the levels of miR-222-3p, miR-486-1-5p, miR-486-2-5p, miR-155, miR-136, miR-494 and miR-495 are up-regulated in exosomes from PE patients [152,153,154,155]. In addition to the expression regulation, miRNA SNPs have been shown to be associated with PE susceptibility, such as the polymorphism of miR-152 rs12940701, [156], miR-146a rs2910164 [157], pri-miR-26a1 rs7372209 [158], miR-155 rs767649 [159] and miR-146a rs2910164 [160] genes.

**Table 2:** Summary of aberrant expression of miRNA in human gestational disorders.

Disorder Name	MiRNAs (Reference)		
Pre-eclampsia (PE)	Up-regulated	proliferation ↓	miR-155 [107], miR-20a [132], miR-16 [135], miR-675 [136], miR-142-3p [138], miR-200p-3b [140], miR-137 [141], miR-146a [143]
		migration ↓	miR-155 [107], miR-431 [116], miR-20b [133], miR-16 [135], miR-125b [139], miR-200p-3b [140], miR-137 [141], miR-146a [143]
		invasion ↓	miR-431 [116], miR-30a-3p [117], miR-20a [132], miR-20b [133], miR-141 [137], miR-142-3p [138], miR-125b [139], miR-146a [143], miR-517a/b [145], miR-517c [145]
		apoptosis ↑	miR-30a-3p [117], miR-29b [134], miR-16 [135], miR-200p-3b [140]
		differentiation ↓	miR-17~92 clusters [146], miR-106a~363 clusters [146]
		other/targets ↓	miR-17 [68], miR-206 [129], miR-210 [131], miR-202-3p [142], miR-155 [144]
		in exosomes	miR-222-3p [152], miR-486-1-5p [153], miR-486-2-5p [153], miR-155 [154], miR-136 [155], miR-494 [155], miR-495 [155]
	Down-regulated	proliferation ↑	miR-378a-5p [101], miR-376c [147], miR-335 [149], miR-126 [150]
		migration ↑	miR-378a-5p [101], miR-3935 [124], miR-376c [147], miR-126 [150],
		invasion ↑	miR-378a-5p [101], miR-3935 [124]
		apoptosis ↓	miR-335 [149], miR-125a [151]
		differentiation ↑	miR-126 [150]
		other/targets ↑	miR-195 [148]
		in exosomes	miR-153-3p [152], miR-653-5p [152], miR-325 [152]
Fetal Growth Restriction (FGR)	Up-regulated	oxidative stress ↓	miR-199a-5p [161]
		other/targets ↓	miR-424 [162], miR-519a [163], miR-1323 [164], miR-516b [164], miR-515-5p [164], miR-520h [164], miR-519d [164], miR-526b [164]
	Down-regulated	other/targets ↑	miR-518b [163], miR-16 [165], miR-21 [165]
Gestational Diabetes Mellitus (GDM)	Up-regulated	other/targets ↓	miR-98 [166], miR-518d [167], miR-144 [168]
		in exosomes	miR-122-5p [26], miR-132-3p [26], miR-1323 [26], miR-136-5p [26], miR-182-3p [26], miR-210-3p [26], miR-29a-3p [26], miR-29b-3p [26], miR-342-3p [26], miR-520h [26], miR-330-3p [169]
	Down-regulated	proliferation ↑	miR-296 [170], miR-96 [171], miR-137 [172]
		migration ↑	miR-296 [170]
		invasion ↑	miR-296 [170]
		in exosomes	miR-125b [168], miR-516-5p [173], miR-517-3p [173], miR-518-5p [173], miR-222-3p [173], miR-16-5p [173]
Early Recurrent Miscarriage	Up-regulated	apoptosis ↑	miR-365 [174], miR-149 [175]
		other/targets ↓	miR-4497 [176]
	Down-regulated	proliferation ↑	miR-155-5p [177]
		apoptosis ↓	miR-155-5p [177]
Preterm Birth (PTB)	Down-regulated	other/targets ↑	miR-338 [178], miR-29b-3p [179]
Small-for-Gestational-Age (SGA)	Down-regulated	other/targets ↑	miR-21 [165], miR-16 [165]

### 6.2. Fetal Growth Restriction (FGR)

Aberrant expression of miRNAs has been also observed and verified in other gestational disorders, including FGR. FGR is defined as the failure of a fetus to reach to growth potential and the occurrence of FGR is closely associated with multiple factors, such as maternal factors, fetal factors, placental factors as well as external factors [180]. The previous studies have revealed that the increased levels of some miRNAs are associated with the pathogenesis of FGR, such as miR-424 [162], miR-199a-5p [161], miR-519a [163], miR-515-5p, miR-1323, miR-520h, miR-519d, miR-516b and miR-526b [164], whereas the expression of some miRNAs is markedly reduced in infants with FGR, including miR-518b [163], miR-16 and miR-21 [165]. Additionally, miRNA SNPs, such as miR-146a G/C rs2910164 variation [181], exert inhibitory effects on fetal growth. However, a previous report showed that there is no significant difference in exosomal miRNA expression between women with FRG and controls [152].

### 6.3. Gestational Diabetes Mellitus (GDM)

GDM is characterized as glucose intolerance (any degree) with onset or first recognition during human pregnancy, which has emerged as one of the complications with high incidence during human pregnancy [182]. Several studies have demonstrated that miRNA expression is also associated with GDM occurrence, and aberrant expression of miRNA in human placenta gives rise to the pathogenesis of GDM, such as miR-296, whose expression is improperly down-regulated in placenta with GDM in clinic and affects human placental development through the alterations in migration and invasion of trophoblast cells by regulating HIF3A [170]. Similarly, the expression of miR-96 is observed to be suppressed in GDM samples and the down-regulated miR-96 exerts protective effects on pancreatic β-cell function via regulating PAK1 in GDM [171]. In addition, high glucose inhibits HTR-8/SVneo cell viability and proliferation through regulation of miR-137 expression, which in turn modulates the downstream PRKAA1/IL6 axis [122,172]. Conversely, up-regulation expression of miR-98 in human placental tissues with GDM is closely associated with the global DNA methylation status through targeting MECP2 [166], and the increased expression levels of miR-518d in the placentas of females with GDM are negatively correlated with the expression levels of PPARα protein [167]. Similarly, it has been found that the expression of exosomal miRNAs is also altered between women with GDM and normal pregnancy, suggesting the changes in the expression of certain miRNAs could be used for diagnosing GDM [183]. For example, miR-125b and miR-144 are always dysfunctional in the circulating exosomes and placenta of women with GDM [168], and the expression of miR-516-5p, miR-517-3p, miR-518-5p, miR-222-3p and miR-16-5p is down-regulated in exosomes from patients with GDM, particularly at the third trimester of gestation [173]. In contrast, the expression of miRNAs, such as miR-330-3p [169], miR-122-5p, miR-132-3p, miR-1323, miR-136-5p, miR-182-3p, miR-210-3p, miR-29a-3p, miR-29b-3p, miR-342-3p and miR-520h [26], is elevated in exosomes from GDM patients. Intriguingly, recent studies have also indicated that miR-binding SNPs such as SLC30A8 rs2466293 and INSR rs1366600 increase GDM susceptibility [184,185]. Similarly, Wang et al. reported that SNPs loci rs2466293, rs1366600, and rs1063192 located in the miR-181a-2-3p, miR-106, and miR-323b-5p binding regions, respectively, all increase the risk of GDM [185,186], whereas the C allele of miR-27a rs895819 could reduce the risk of GDM [187].

### 6.4. Other Disorders

In addition to PE, FGR and GDM, miRNAs such as miR-365, miR-4497, miR-155-5p and miR-149 are found to be associated with early recurrent miscarriage [174,175,176,177]. Moreover, in placenta of women with PTB, the expression of miRNAs is found to be altered, such as miR-338 [178] and miR-29b-3p [179], and interestingly, a comparison analysis study of the exosomal miRNA profile between term group and PTB group revealed a total of 173 miRNAs with significant alterations across gestation [188,189], indicating the exosomal miRNA expression pattern may predict the risk in PTB. Furthermore, the down-regulated expression of miR-21 and miR-16 in human placenta is shown to be predictive of SGA status [165], and a latest study reported that SGA children have a distinct exosomal miRNA expression profile compared with appropriate-for-gestational-age children [190], also providing the evidence that exosomal miRNAs may serve as novel biomarkers for SGA. On the other hand, SNPs recently have also been shown to be associated with these disorders. For example, polymorphisms in miRNAs such as miR-27a, miR-449b, miR-150 and miR-1179 are associated with recurrent miscarriage [191,192], while rs1537515 located in the binding sites of miR-1224-3p and miR-3150-5p increases the PTB susceptibility [193], and similarly, miR-1304-3p rs1537516 increases the risk of PTB [193]. Moreover, Silva et al. recently reported that reduced miR-146a rs2910164 presents increased frequency in SGA children [194].

## 7. Perspectives

Detection of specific miRNAs in different gestational stages and in the maternal circulation provides with the possibility that miRNAs may be used as pregnant-related biomarkers to monitor the status of human normal pregnancy and potential gestational diseases in clinic. However, quantification of miRNAs, particularly the exosome miRNAs, is challenging due to their small amount. Thus, it is important to improve current experimental methodologies and to hopefully make comparisons of the experimental data obtained with available databases on line as well as with in silico prediction models to achieve a complex view on the role of miRNAs in different gestational diseases and to further discover suitable biomarkers. Owing to the great work in past decades, we have obtained a better understanding of miRNAs in human pregnancy. Nevertheless, despite the exciting results on miRNA research in human placenta, our current knowledge on how the thousands of miRNAs contribute to human placental development and function in pregnancy processes is still very limited. Studies in the future are in need to continue to search for novel miRNA candidates, to investigate the regulation pattern of those miRNAs expression and to further decipher the molecular mechanisms underlying their regulation in human placenta.

## Data Availability

Not applicable.
